# The *Țigani* Community Adaptability to Changes in Rural Romania and the COVID-19 Impact

**DOI:** 10.3390/ijerph182010622

**Published:** 2021-10-11

**Authors:** Mihai Voda, Andrei Murgu, Constantin Adrian Sarpe, Steven M. Graves, Calin Avram

**Affiliations:** 1 Geography Department, Dimitrie Cantemir University, 3–5 Bodoni Sandor St., 540545 Targu Mureș, Romania; 2 Faculty of Law, Lucian Blaga University of Sibiu, 10 Victoriei bd., 550024 Sibiu, Romania; abmurgu95@gmail.com; 3 Romanian National Waters Administration, 33 Samuel Koteles St., 540057 Targu Mureș, Romania; adrian.sarpe@dam.rowater.ro; 4 Department of Geography and Environmental Studies California State University, Northridge, CA 91330-8249, USA; steve.graves@csun.edu; 5 Biostatistics Department, George Emil Palade University of Medicine, Pharmacy, Science and Technology, 38 Gh. Marinescu St., 540139 Targu Mureș, Romania; calin.avram@umfst.ro

**Keywords:** *Țigani*, adaptability, sustainability, social environments, COVID-19, cultural identity

## Abstract

Romanian rural villages are struggling to survive present times when youngsters leave for a better life in the city while elders work the land like a hundred years ago. Our paper integrates human environments research with public health preparedness, presenting the *Țigani* (Gypsy/Roma) ethnic group from rural Romania as an example to the world. The future security of mankind will require a new understanding of the human place in its environment. That will lead to a new society, not the most powerful or intelligent, but the one that is more adaptable to changes, with sensitive and interconnected community members. Therefore, the *Țigani* ethnic group that fought for its rights and flourished despite unfavorable odds, including the recent COVID-19 pandemic, represents the best example for a new world that prioritizes humans, promotes health and wellbeing, facilitating innovation and transformative networks environmental integration. This research attempts to quantify the *Țigani*′s unique attributes that helped their communities survive and made them more adaptive to change. Always marginalized, they identified the other ethnic groups’ weaknesses to penetrate the villages and learned to use the smartphone apps to communicate, for their trades, coppersmith, metal roof tiles and drainage systems. Our research was based on Geographical Information System, Microsoft Power Bi analytics data visualization tools and statistical analysis with SPSS V20 to demonstrate what enables their flourishing and what resistance they face locally. We argue that the *Țigani*′s intense social cooperation, strong sense of family, community and mutual assistance helped them to fight COVID-19, generating their significant adaptability to the societal changes and their power to keep intact their cultural identity. The results show how the constant growing *Țigani* population changed and may change Romania′s rural environments in the future.

## 1. Introduction

Today’s Romanian rural villages face considerable difficulties because so many youngsters leave seeking jobs in Western Europe. They leave an increasingly elderly population working the land much like it was done hundreds of years ago. In recent years, many Romanian rural workers have migrated to the Western European countries in search for employment. Millions have left for Italy, Spain, Germany, or the UK looking for better wages. Romania’s emigration growth rate was recently second only to war-torn Syria [1]. The unusual Romanian exodus has been generated by significant inequalities in wealth, income, and opportunity [2]. Few Romanian migrants intend to return to their villages. Meanwhile, in many of these same villages, *Țigani* families (Gypsies/Roma) remain, and often thrive despite the economic challenges. Some *Țigani* groups, such as the Gabors in Crăciunești or the Căldărari in Buzescu have even prospered in recent years, building mansions with pagoda style roofs and colonial porches [3,4]. This paper offers some analyses of the factors attributable to the success of the *Țigani* people, despite the challenges created by their history. We consider that the legacy of discrimination and bias against members of this group has likely wrought within them an ability to adapt to rapid changes in technology and economic circumstances that has served them well, even during the recent global pandemic.

For readers beyond Romania, an explanation of terminology used to describe our community of interest is in order. In popular parlance the label “Gypsy” has been widely applied to this group. The term Roma has been advanced by the United Nations and Council of Europe. Klimova-Alexander (2005) [5] explained how Roma elites are establishing a new identity and constructing imagined communities based on the United Nations’ and the Council of Europe’s promotion of the term Roma. We use the term *Țigani* instead. According to Cherata (2014) [6], the word *Țigan* is used under the form *o tsigano* in the Romani language as in “San Rrom Tsiganiako!” meaning you are a true, genuine gypsy (i.e., you are one of us, a member of our Gypsy community). *Țigani* is a unique noun that shares some of the stereotypical associations tied to the English term “Gypsy” which is less precise. [7]. As Vamanu and Vamanu (2013) [8] recommended, the Romanian term *Țigani* marks the specificity of the Roma in Romania. We use it in signifier of respect to the “tradition keepers”. The use of this appellation also signals that we respect the use of the indigenous terminology and seek to disassociate from negative stereotypes that burden other terminologies [9].

In a time marked by crises of immigration, intense international scrutiny of racial and ethnic inequalities, many of which have become magnified by the COVID-19 pandemic, it is worth examining the *Țigani* in Romania. The history of the *Țigani* is one of the most well-known, ancient and troublesome migration stories; one fraught with an unusual pathway toward national and ethnic cultural integration. Romania presents an excellent case study in the complex processes associated with cultural and economic integration among people intensely focused on maintaining both a cultural identity while seeking economic prosperity. Their experience in Romania may present some lessons for other regions and other identities, such as the Amish in the United States, or other immigrant groups to Europe who are determined to resist full cultural integration [4,10,11,12,13].

We argue that the *Țigani* have curated a certain kind of economic pliability that we will call adaptability. This characteristic, we believe, is a product of their difficult history of persecution and migration. Their willingness to adapt to rapid economic change competes against their openly professed desire to protect their cultural identity. The findings presented in this article address research questions that seek to uncover the *Țigani* community′s adaptability. This is a somewhat unique contribution to the literature because it recognizes one of the commonly overlooked attributes of the *Țigani* community. Although they have been victims of bias, we posit they are not as vulnerable as much of the literature suggests, especially in the geographic context provided by Romania, especially in and around Mureș County, a region of the country marked by generations of cultural heterogeneity. Their ability to adapt to rapid economic changes since the 1990s have allowed them to overcome multiple challenges, including the recent pandemic, are presented as evidence of their ability to navigate novel economic situations even while they maintain many of their traditional cultural practices.

Our approach facilitates an analysis of entire *Țigani* Geosystems where the digital revolution empowers the place-based activities extension for the wellbeing of all inhabitants. Domin (2017) [14] revealed the *Țigani* community′s central cultural figures, such as Ioan Budai Deleanu, the writer of *Țigani*ada (an epic piece of Romanian literature) or Anton Pann (with Căldărari origins), one of the first major interpreters of Romanian folklore. Our approach includes the notion of Geosystem. The geosystem is a geographical system that prioritizes humans, viewed like a living organism which generates welfare for its creators if they have the capacity to control the matter, energy, and information fluxes to constantly maintain it in a dynamic equilibrium state. The extension of each Geosystem depends on the human community′s egregore that defines the geographical space. The people′s collective group mind, its egregore, represents the inner quintessence of each functional Geosystem with social connections and collaborative actions [15]. The COVID-19 pandemic has highlighted inequalities, but also made evident this community’s response strategies.

Our research capitalized on issues of mobilities, health and the value of mutual aid, each an aspect of *Țigani’s* misunderstood attributes: interdependency. As members of their community, they are obliged to help and support each other in a way that highlights near folk culture-like characteristics, in the age of the internet. Further, we argue that more localized indigenous Geosystems with *Țigani* groups exhibit greater resilience and adaptability. This approach reflects the most important features for the critical functionality of the individual Geosystems [16]. There might be a large research literature about the Roma/Gypsy [3,4,5,6,7,8,9,10,11,12,13,14], but few of the studies offer praise for the *Țigani* nor have they asked the type of questions related to adaptability and the *Țigani’s* peculiar system of community. Our research aims to develop new contextualized theories with the objective of discovering general patterns that hold for a community [2]. The *Țigani* adaptability is generated by their distinct attributes: identity (proud to be different), a strong bond to the collective which makes them interconnected–interlinked–interdependent.

Our goal is to analyze what enables the *Țigani* to flourish economically as a distinct ethnic group that accepts the value of peaceful coexistence with Romanians, Hungarians, and Saxons in Central Romanian villages. The study contributes to an understanding of why a considerable number of *Țigani* communities are not leaving Romania for a better life in the western European countries. Our approach makes it possible to analyze entire rural Geosystems by emphasizing an integrative approach that maintains peoples’ key position in the geographical place. The study contributes to the understanding that the sustainable management of local resources represents a lifelong learning environment where poor *Țigani* communities have access to the new technological advances. We also show the resistance the *Țigani* groups face locally and how the human values and social networks are empowered by the creative use of smartphone applications. They learned to use the smartphone apps for their trades, metal roof tiles and drainage systems, coppersmith or even business development.

We hope that in addition to empirical observations, this article will make a theoretical contribution as we develop the concept of adaptability from our analysis of the *Țigani* responses to economic and cultural change.

## 2. Materials and Methods

We used Geographical Information System (GIS) (ESRI, Redlands, CA, USA), Microsoft Power Bi (Microsoft Power Platform) analytics data visualization tools and SPSS V20 (IBM Corporation, Armonk, NY, USA) statistical analysis with data collection based on publicly available surveys and geospatial datasets to observe and catalog what seems to have enabled the *Țigani* community’s culture of economic–cultural adaptability to changes as well as the resistance they face in the local context.

The first step in our research was to collect and map data from the Tempo Online database of the National Institute of Statistics (1930, 1992, 2002, 2011 national censuses) [17]. The mapped data showed an increasing number of *Țigani* communities in rural Romania and the migration patterns from the Danube River to the North and West with a preference for Central Transylvania in Mures County. Our analysis of the settlement pattern also revealed that the largest *Țigani* communities formed in mixed Romanian/Hungarian/Saxon villages and in deserted Saxon villages. We mapped the pattern of *Țigani* migration based on the national censuses data [17], compared that to the literature written about *Țigani*/Roma/Gypsy migration and found they do match [3,4,5,6,7,8,9,10,11,12,13,14]. The GIS analysis confirmed the *Țigani* groups migration from South to the North through Central Romania and then to the West towards Hungary. They simply followed the main communication routes and preferred settlement in the mixed Romanian-Hungarian villages in Mures County where many successfully integrated into the local fabric of these villages. We explained how they occupied deserted Saxon villages and the socioeconomic aspects of their daily lives. The map helped us narrow our attention upon the specific *Țigani* groups that have been able to penetrate and adapt in the rural villages, largely in Transylvania. However, how were they able to better adapt in some villages compared to others?

In June 2017, the first author started to collect information from Neaua village inhabitants about the *Țigani* groups who lived there. Five non-structured interviews were performed with the local Priest (male 40 years old), the Neaua village Major (male, 59 years old) and three *Țigani* elders, heads of their families, each one over 60 years old, males. The results were presented at a conference organized by the Geography Department of Warsaw University on 25 September 2017. Neaua′s *Țigani* were residentially integrated, but strongly segregated in Sansimion, Vadas, Ghinesti and Rigmani, neighbor villages under Neaua administration.

The second research step was to gather information from The Romanian Institute for Research on National Minorities who provided data on a number of the 1387 rural villages that have a *Țigani* community. The database is publicly available, offering information about rural Romania segregation and living standards [18]. With the Microsoft Power Bi analytics data visualization tools, we observed the errors of segregation index which relates to groups of small villages, administratively united in a commune. Therefore, we were able to correctly analyze the Romanian National censuses data [17] which showed that numerous *Țigani* ethnics live in mixed Romanian-Hungarian villages in Mures, the county with the largest *Țigani* population in Romania. To understand why they were able to better adapt in mixed villages compared with ethnically homogeneous ones, we determined the villages with *Țigani* target groups based on the national censuses data analysis results. The selected rural villages have similar natural settings, socioeconomic level, and cultural characteristics. We structured them in categories based on egregor strength and ethnic admixture to show *Țigani*′s ability to fight poverty, their significant adaptability to the societal changes and their power to keep intact their cultural identity despite unfavorable odds. The first category included Hungarian communities with a weak egregor such as Neaua and Crăciunești, invaded by traditional Gabori. In the second category we analyzed the tight-knit Romanian/Hungarian villages with a strong egregor where the residents never sold land to traditional or modern *Țigani* families such as Hodac and Chibed. The third category is represented by the numerous villages with mixed Romanian-Hungarian population and many traditional Gabori and modern Rroma/Roma people such as Bagaciu, Bahnea, Band, Cristesti, Sanpaul, and Vanatori.

Between June 2020 and May 2021, all the authors explored and collected data from specific rural *Țigani* communities, observing the traditional Căldărari coppersmiths from Brateiu village, the Gabors from Crăciunești or the ethnic mixed villages where *Țigani* groups thrived such as Bagaciu, Bahnea, Band, Cristesti, Sanpaul, and Vanatori. A total of 15 non-structured interviews provided information from the *Țigani* groups representants who were generally over 45 years of age, males, leaders of their groups. We made close observations of the differences and similarities between different *Țigani* groups (*neams*). The process of entering the *Țigani* groups was facilitated by the common geographical background with two of the authors, who live among the *Țigani*—in ethnically heterogenous villages in Transylvania, allowing them many years of observation, interaction and that forms part of the informal data collection that informs the research. Our first and second authors live in mixed villages, with *Țigani* neighbors alongside Romanians, Hungarians and even Saxons.

The next step of our research was to collect data from the Mureș Public Health Directorate (March 2020–February 2021) [19] for a COVID-19 analysis to prove the adaptability of *Țigani* community to health emergencies, based on their unique attributes. A cross-sectional analysis was performed with data on the number of SAR-COV 2 (COVID-19) infections collected from the Mureș Public Health Directorate [19] between March 2020 and February 2021. To identify the normality of the data we performed the Shapiro–Wilk test. Due to the non-parametric datasets, we applied the Mann–Whitney test.

Finally, we developed theories about the *Țigani*′s unique attributes that seem to be generating their adaptability, constantly comparing our findings against the literature [3,4,5,6,7,8,9,10,11,12,13,14] and the results of our direct observational activities in June 2017 and between June 2020 and May 2021. Living among the *Țigani* community enabled us to observe closely the behaviors of the *Țigani* and listen to their narratives regarding their motivations, hopes and plans. The close personal experiences with Mures County *Țigani′s* reality allowed us to observe what they do rather than what people say they do, emphasizing the ethical advantage and relevance of moderate participant observation in offering authentic data [20,21]. Our participant observations between 2020 and 2021 permitted *Țigani* community members to speak for themselves. This is how we understood that *Țigani* groups are proud to be different, family interdependent, tradition keepers and that they value basic education.

With this innovative approach, the investigation of geographical space gains new dimensions and applicability potential in terms of Geosystem′s unique attributes recognition and validation, for the transformative networks’ inclusion. This is how, in addition to empirical research and practical implications, this study will make a theoretical contribution and develop the concept of adaptability.

## 3. Results

The availability of environmental datasets facilitated the assessment of unique attributes connections inside the functional *Țigani* Geosystems, with the goal of discovering general patterns that hold for a community. We used the map of the *Țigani* populations to select locations for observation and validate their invasive movements.

What defines the functional *Țigani* Geosystem? What are the *Țigani* communities’ unique attributes?

A brief history of *Țigani* is necessary to understand that the functionality of a *Țigani* Geosystem is based on their inter- and intra-family interactions [6,9,11,22,23], strong sense of identity [4,7,9,12] and traditions [3,4,8,14]: the traditional community′s unique attributes.

According to the literature [3,4,5,6,7,8,9,10,11,12,13,14,22,23,24,25], when the *Țigani* migrators first arrived from the South in the Romanian Principalities it was the during the late XIV century. Due to their skin color and cultural characteristics, they were initially seen as enemies and enslaved [22,23]. In the Central and Western Europe, the first *Țigani* clans were initially tolerated, treated with curiosity and empathy, not enslaved. However, due to their living style based on non-Christian doctrine, which locals often considered witchcraft, their embrace of activities like fortune telling and a reputation for theft, they were marginalized, and a long history of discrimination and adaptability to bias started. Many areas in Western Europe took measures against *Țigani* groups to prevent permanent residency. Rarely were they integrated or even tolerated in the more developed western European realms, especially where established trade guilds and their resultant high-quality trade goods discouraged competition from *Țigani* craftsmen. The Romanian Principalities, where a predominantly agrarian economy lingered, accepted more quickly the rudimentary products of *Țigani* craftsmen thereby encouraging at least a measure of economic integration with the *Țigani*.

**Figure 1:**
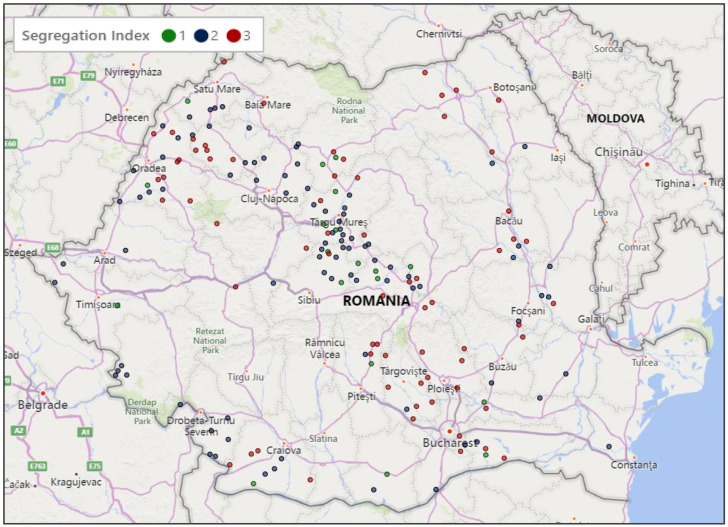
Segregation index in Romanian villages with greater than 15% *Țigani* population.

Comparing the historical narrative from existing literature [3,4,5,6,7,8,9,10,11,12,13,14,22,23,24,25] with our GIS map (Figure 1), it can be argued that the integration of the *Țigani* process repeated itself many times over across Romania but was most pronounced in ethnically heterogenous villages where the heterogeneity seems to have created space for the *Țigani* to perhaps avoid the type of discrimination that forced them to continue migrating elsewhere. Figure 1 (below) shows their migration path from the Danube River, the route of migration and the resultant density of *Țigani* settlement in the Transylvania region, especially in Mureș County. Many of those able to merge into the villages that were majority Romanian declared themselves Romanians in the 1930, 1992, and 2011 censuses [17]. The same situation was observed in Transylvanian villages where those accepted embraced the majority′s Reformed Hungarian religion (Erdélyi Református) and ethnicity.

It is important to notice that the *Țigani* were considered an inferior ethnic group by other ethnicities, but evidence also suggests that in much of Romania, the *Țigani* always had a place in the local economy. Unlike elsewhere, they were never actively persecuted, evacuated, or deported. Moreover, in Transylvania, they had the liberty to travel and settle wherever they wished on the “king′s land”. During the XV and XVI centuries, *Țigani*′s privileges were maintained: each *Țigan* had to pay one florin for taxes each year, compared with the two florins tax paid by the regular paysans, settled in the landlords’ villages [23,25].

Into the 20th century, many Romanian rural villages retained a rudimentary agrarian economy, where the products of *Țigani* craftsmen continued to be useful. When the communist collective enterprises called Cooperativa were destroyed during the 1990s and land was divided in small, often non-contiguous, private parcels that were generally inefficient for agriculture, farming was rarely profitable. Inefficiencies were compounded because the new landowners had little capital for tractors and other mechanized farm equipment, requiring continued use of horses or oxen to pull plows and harvesters.

The first groups of our participant observation activities between 2020 and 2021 were conducted among the traditional *Țigani* in Neaua and Crăciunești, villages with a weak Hungarian egregor (the collective mind allows people to sell land/houses to *Țigani*). Our observational activities were done in 19 similar Transylvanian natural settings, providing access to *Țigani* community, enabling us to observe what the *Țigani* do so that we can compare their behavior to what other ethnics (Hungarians, Romanians and Saxons) say they do.

**Figure 2:**
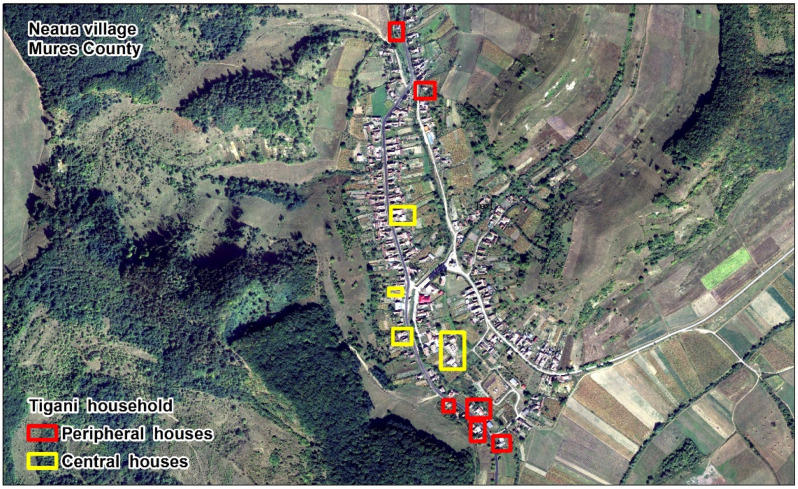
Neaua village peripheral and central Țigani households [26].

Although the *Țigani* groups were and continue to be discriminated against by some, even in Romania, they are tolerated in many peripheral areas on the edges of villages. Although marginalized, the *Țigani* were quick to identify the needs and economic shortcomings of other ethnic groups within the villages as they established their own economic and cultural niches. For example, the traditional *Țigani* community from Neaua, a village with a weak Hungarian egregor, has grown significantly in the past 50 years. Growing from a single couple living at the periphery of the village to a dozen family groups who have slowly moved towards the central areas (Figure 2). They have bought houses in the center of the village from ethnic Hungarians, many of whom have left for a better life elsewhere.

Our analysis finds that nearly all villages tolerate at least small *Țigani* groups in the settlements′ peripheral areas, but only the ethnically mixed villages accommodate large numbers of *Țigani*. Some *Țigani* families consider themselves Hungarians and go to the Reformed Church (Reformată) church, others are declared Romanians and go to the Orthodox church. The unique Gabors are neo protestant, interacting with the first author every weekend when he visits his wife′s parents in Neaua village. In the ethnically homogenous villages *Țigani* appear to be less welcome. Take for example, the compact Hungarian village of Chibed, where a collective, defensive sense of community prevents local Hungarians from selling property to *Țigani*. A similarly strong unspoken collective agreement, a product of the local egregor, characterizes the behavior of Romanian villagers in Hodac.

In ethnically diverse Mureș County with 277,372 Romanians and, 200,858 Hungarians, the *Țigani* have found the most tolerant, if not receptive locations to settle. Today it has the highest number of *Țigani*. In the 1930 census there were 18,878 *Țigani* registered in Mureș, a number that almost doubled in 1992 to 34,798, and 46,947 people in 2011 [17]. Centrally positioned on the main route to the Western Europe, Mureș County has a considerable number of ethnically heterogenous villages where *Țigani* groups have thrived such as Bagaciu, Bahnea, Band, Cristesti, Sanpaul, and Vanatori. They used the main route that follows Prahova River to cross the Carpathian Mountains, constantly moving through Brasov-Mureș-Cluj-Bihor counties towards Hungary and Slovakia.

The population of *Țigani* has blossomed, though exact figures are difficult to find. According to the Council of Europe, 1.85 million Roma (*Țigani*) live in Romania [24]. The 2011 census declared that only 621,573 people identified themselves as Rroma-*Țigani* [17]. We think that European Council assigns too many Romanians (1.24 million) with unknown ethnicity to the “Roma” ethnicity, while ignoring the concerns of experts such as Surdu (2019) [12] who emphasized the rejection of ethnic categorization, and Cernat (2020) [13], who points out the important methodological changes introduced by 2011 census to count and categorize Romanian citizens who are working in other European Union countries.

Traditional *Țigani* continue to manage to avoid the need for much formal education. They get by and still maintain a traditional way of living, conserving their memes as units of cultural transmission [27]. The Gabors, for example, have their own law, preferring to develop some sort of home schooling which they consider the school of life; not altered by “the system”. They consider their kids free and insist they will not become servants. As Olivera (2019) pointed out, they are also the keepers of traditional clothing and should be considered natives of a special kind [9]. Their women wear colorful skirts, aprons, silk headscarves that cover their long-pleated hair. Their mustachioed men typically wear black felt trousers, a leather waistcoat, a felt jacket and a black hat. According to Constantinescu (2006) [28], the Gabors represent the most traditional Gypsy clan (*neam țigănesc*) of *Țigani* community, yet they have proven to be very skilled businessman, versatile and ingenious, always adapting to different environments or societal changes. They trade beakers and tankards with the Cărhari, who are skilled coppersmiths and livestock raisers who respect ancient traditions by collecting silver prestige objects as proof of ethnic identity and history [29].

Although they have minimal education, the traditional *Țigani* communities (popularly called “Gypsy”) such as the Gabori, Căldărari, Spoitori, Caramidari, Florari and Argintari tend to be fluent in their native language of Romany and generally possess excellent entrepreneurial expertise. The more modern *Țigani* (also called Rroma/Roma people) generally have only basic or no Romany language skills. Rroma, Vatrashi and Lăutari represent successful *Țigani* groups (*neam*). They tend to be highly educated and politicized. Ten years ago, the *Țigani* community members would not have thought about any smartphone or social media opportunity to promote their businesses, but today′s technology makes it possible for anyone to be on display instantaneously for free.

The Romanian government rights-based policies have allowed them to move and live on public land, build houses without pre-approved construction plans, to register themselves and their children with the local administration office to improve their well-being, which permits them to benefit from free education and medical services.

Moreover, in the Hartibaciului Valley villages, local *Țigani* had the opportunity to move into government houses after the massive migration of local ethnic Saxons (Germans) to Germany in the 1990s [23]. Saxon houses were bought by the Romanian authorities from each out-migrating family, so the *Țigani* settlers faced little resistance locally. *Țigani* regularly find temporary work, often getting paid daily by the wealthier householders. The less successful *Țigani* clean village′s streets, collect trash, become logging workers or community cattle shepherds. The Caramidari are brick makers, and the Rudari (former gold and coal miners) make willow brooms, brushes, and baskets [28]. The successful *Țigani neam* represented by Argintari are silversmiths and goldsmiths, the Gabors, Căldărari and Spoitori are coppersmiths, making buckets, stills, cauldrons, tin roofing material, water drains and gutters. The Florari are flower sellers and the Lăutari excels in music making. All *Țigani* communities have access to the new technological advances, benefiting from high-speed internet through desktop computers, laptops, tablets, or smartphones. They learned how to promote their traditions on social media such as Facebook and YouTube, communicate on WhatsApp, sell their services and products on Olx web platform.

Always adapting to the societal changes, *Țigani* started to explore other countries when Romania joined the European Union (EU) in 2007. Some Cărhari migrants became beggars in Italy and France [29]. Some of the long-bearded Căldărari elders from Brateiu found they could earn more money asking for charity in front of western churches than by creating copper artifacts back home. Unlike their fellow Romanian citizens, who were generally considered reliable workers, and therefore encouraged to bring their families as well, the *Țigani* groups were marginalized in much of Europe and sent back [30]. Many western European cities worked to discourage *Țigani* migration. Rather than attempt to integrate *Țigani* communities into their society, the EU, instead offered international support to the Romanian rural communities where they were coming from to encourage them to stay in Romania. Unlike many Romanians, *Țigani* are not leaving Romania in vast numbers for a better life in the western European countries. The Romanian ′exodus′ has been generated by the important income and opportunity differences but the *Țigani* community managed to use its social connections and collaborative actions to survive and thrive [2]. They are not vulnerable at all. They are one of the most adaptable communities, capable of facing any challenges. The following COVID-19 analysis is proving the adaptability of the *Țigani* community to health emergencies, based on their unique attributes.

COVID-19 has aroused previously ignored cultural geographical inequalities. Our research capitalized on issues of health and the value of mutual aid, which is related to one of *Țigani*′s unique attributes, interdependency. As members of their community, they are obliged to help and support each other. As Millan and Smith (2019) [31] observed in UK and Ramos-Morcillo et al. (2019) [32] in Spain, about the Gypsy concept of health, we analyzed in Mures county, using the pandemic to emphasize the great capacity of *Țigani* community to easily adapt to different challenges, including COVID-19. Millan and Smith (2019) [31] suggest that health authorities should routinely and systematically monitor Gypsy Roma and Traveller people′s health. As Ramos-Morcillo et al. (2019) [32] observed on Spanish Roma communities, healthcare systems should merge culture and health care. The Romanian *Țigani* community prioritize optimism and happiness in a state of good health which could be leveraged by health officials. When the physical implications of COVID-19 were visible, the need to act at the community level to impose policies was evident. It is important to notice that in the traditional *Țigani* communities, such as the Gabors from Crăciunești, when one is sick, others are quick to give moral support. They go in groups to the hospital and stay together all the time. The mutual assistance commitment comes from the spousal control which generates marital alliances between families, social and economic collaboration among community members [28].

### Statistical Analysis Results

According to the last census, Mures county had a population of 550,846 inhabitants. Over half (276,773) live in cities and towns and the remainder (274,073) live in the county’s villages. The distribution by ethnicity in the rural villages is as follows: ethnic Romanian population 127,696 (47%), ethnic Hungarian population 103,157 (38%) and *Țigani* 33,692 inhabitants (12%). The *Țigani* are not evenly dispersed, but rather concentrated in localities where they often constitute over 25% of the population of that locality.

During the COVID-19 pandemic, the Crăciunești village with approximately 30% *Țigani* ethnics was hard hit during the first reporting period. That village alone accounted for 25% of the total number of COVID-19 cases in Mureș County. After becoming aware of how community spread worked, the *Țigani* quickly adopted the basic rules of social distancing, mask wearing and hands washing, and the number of infections decreased. In Band village, during the third reporting period, there were a higher number of infections, yet they represented only 0.5% (out of a total of 9676 infected patients) of the total number of infected people in Mureș County, compared to 26% of the number in the first period for Craciunesti village (Table 1).

In Table 2 we identified the number of infected patients over the four reporting periods. Data from villages were separated into two classes: those with over 25% of *Țigani* and villages with less than 25% *Țigani* people. In the localities with over 25% *Țigani* the following distribution is as follows: total population is 44897, the *Țigani* number 13,529 (30.13%), Hungarians number 12,713 (28.13%) and ethnic Romanians number 16,221 (36.12); the remaining 5% come from other ethnic groups. A more pronounced infection is observed in the first reporting period, between March and May 2020, after which the number of infected people decreases. From this data table we can identify the number of infections (N), which in the two categories in the first reporting period was higher in villages with more than >25% *Țigani* (Table 2).

**Figure 3:**
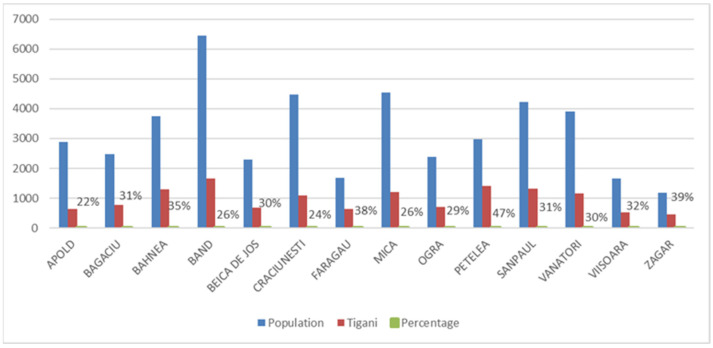
Mureș County villages with more that 25% of *Țigani* population.

In the first reporting period, the average percent of infected people calculated from the number of infections in villages with more than 25% *Țigani* (Figure 3) is higher than the average of villages with less than 25% *Țigani* (9.46 compared to 3.61). In the third reporting period in localities with many *Țigani* the data are more compact (SD = 14.56). On the other hand, in the localities where the *Țigani* are fewer, the SD is higher than the average, which means that in those localities there are very big differences between the minimum number and the maximum number of infections.

To compare the data from the two categories (Villages with more than 25% *Țigani* vs Villages with less than 25% *Țigani*) we applied the Mann–Whitney test due to the fact that the data did not pass the Shapiro–Wilk normality test. It can be stated, based on the result of the applied test, that we have no statistical significance for the reported periods (the significance level chosen for our test, usually 0.05) (Table 3).

The results show that *Țigani* community′s high number of COVID-19 infections in the first wave slowly decreased as they started to learn from observation and experience of their villages’ cohabitants.

## 4. Discussion

Tesfay [4] claims that the sense of family and community helps preserve the identity of traditional *Țigani* while Berta [29] points out the importance of prestige objects as markers of ethnic identity which is not entirely negative, according to Olivera [9]. We agree with Tesfay [4] and Berta [29] about the strong interconnection between families–communities, markers and sense of collective identity but we feel that Olivera is too dependent on optimistic data, disregarding the general concern of other experts who consider the *Țigani* collective identity as being generally negative [7,8,30].

Our analysis finds that *Țigani* community adaptability is generated by unique attributes such as identity (they are proud to be different) [4,7,9,12,29] collective mind (strong egregor, they are tradition keepers) [3,4,8,14,29], interconnected–interlinked–interdependent community members (information, communication between individuals/families/relatives) [6,9,11,22,23,31,32,33]. Both traditional and modern *Țigani* value education, being aware of its influence upon their living standards [5,10,22,25,28]. During the COVID-19 pandemic, they were increasingly reliant on alternative means of support including charitable projects and claiming state benefits.

The reason why these findings are important is to acknowledge the ways in which the *Țigani* adapt, which was the initial research question of our study. Our research aimed to develop new contextualized theories with the objective of discovering general patterns that hold for *Țigani* community. They learn from observation and experience; this is why the Căldărari from Brateiu are selling their copper art in Poland or the USA and the Gabors from Craciunesti excel in their trades. A large percentage know how to communicate on WhatsApp where they exchange photos, videos, sell objects or even offer drainage system services on olx.ro. Finally, we found that all of the different *Țigani* groups (or *neam* in Romanian language) present the same unique attributes but the best tradition keepers, the Căldărari and the Gabors are the leaders that will preserve the *Țigani* community qualities, their unique attributes which are fundamental to their adaptability to any societal changes. All these attributes are mentioned in the existing literature [3,4,5,6,7,8,9,10,11,12,13,14,22,23,24,25,28,29,30,31,32,33], but not from our theoretical perspective. The findings presented in this article answer the research question on *Țigani* community′s adaptability. It is the first paper in which they are highly appreciated, emphasizing qualities identified as unique attributes. We are not portraying an invasive *Țigani* community [8,30] that is destroying the European identity of Romanians [7]. We only state that they are not the endangered ones. Their adaptation abilities prevailed even the pandemic. The COVID-19 collected data from the Gabors who live in Crăciunești village, demonstrated the great capacity of *Țigani* community to quickly adapt and overcome any difficult situation, keeping their cultural identity intact. 

This paper adds a new layer to the conventional narrative about the *Țigani* found in the literature [3,4,6,7,9,14,22,23,25,28,29] that tend to characterize the traditional *Țigani* simply: tradition-bound and culturally conservative. While it is evident that many elements of *Țigani* culture embrace traditional modes of behavior, our observations and our analysis of the COVID-19 data suggest that such characterizations of the *Țigani* are incomplete. We seek to add to “adaptable”, “flexible” and “resilient” to the list of characterizations of the *Țigani*. We suggest that their ability to rapidly adjust to changes, particularly those related to the economic changes and medical/health protocols are not especially hindered by their embrace of traditional cultural practices. Indeed, time may prove that the *Țigani* are better equipped than many of their neighbors to adjust to the realities of the post-modern economy and the uncertainties of the 21st century.

The future well-being of humankind will require a new understanding of the human place in its Geosystem. That will determine the formation of a new society, not the most powerful or intelligent, but the one that is more adaptable to changes, with sensitive but proud, communicative, and interconnected community members. Therefore, the *Țigani* community represents the best example for a new world that prioritize humans, promotes health and wellbeing, facilitating innovation and transformative networks integration [2,33].

Further research should have a closer examination to predictions through increased formalization, so this complex process could be understood by the general public. The formal model creation facilitates the identification of all the stages that led to the increased adaptability of the *Țigani* community, but also a forecast of its subsequent evolution [34].

## 5. Conclusions

This article examined the *Țigani* community and their ability to adapt to changes in the economic system in the global context and in Romanian rural villages while keeping much of the cultural traditions intact. Our study highlights the historical migration of the *Țigani* ethnic group from the south to the north and west in a continuous fight for their rights, economic prosperity and residential safety, always adapting to any unfavorable odds. Most of them remained in central Romania where a more tolerant, mixed Romanian/Hungarian population allowed them to settle in relative safety. Previous research revealed the indigenous peoples’ essential role in the protection and preservation processes of their living environments.

The locals’ history, legends and myths confer authenticity to the geographical place, creating the foundation of each Geosystem′s egregor. Communities’ access to the new technological advances can help them learn how to conserve the Geosystem′s unique attributes for their own benefits.

Our analyses reveal their ability to adapt to changes in society which have helped their communities survive and prosper even during the COVID-19 pandemic. *Țigani* learn rapidly from observation and experience. Despite the lack of general school education, they quickly adopted smartphone apps to transmit information, to enhance their trades and promote coppersmith, metal roof or drainage system businesses. That leads to a better communication, another one of their unique attributes. Yet, they remain proud of their traditions, they conserve local memes, always preserving and developing the community′s egregor.

Further, the geospatial datasets from Google Earth confirm that ethnically mixed rural villages are very likely the best suited for the *Țigani* to settle and that the increasing number of *Țigani* is slowly changing these villages’ social environments. We also find the *Țigani*′ s ability to rapidly adapt their cultural practices has allowed them to deal with COVID-19 conditions and the process has been comparable to other ethnicities in Romania despite the lack of general education. The main objective, however, was the continuous rearrangement of the environmental components (focused on human as the central element), in order to reintegrate the multiple elements and to obtain a new way of understanding the human place in its environment. The COVID-19 analysis proved the adaptability of *Țigani* community to health emergencies, based on their unique attributes. They are not vulnerable at all. They are one of the most adaptable communities, capable of facing any challenges.

The *Țigani* community’s adaptability to the societal changes and their power to keep intact their cultural identity provides a compelling example for other, similar communities. Further research is needed on the connections between concepts of adaptability, vulnerability and resilience.

The present study did not examine the conflicts generated by the growing *Țigani* population in Romania. Future research might address inter-ethnic conflict and the relationship between the funding for capacity building and the well-being of the *Țigani*’s groups in rural communities.

## Figures and Tables

**Table 1:** The number of *Țigani* community people infected with SAR-COV2 in Mureș County.

Village	March 2020– May 2020	June 2020– August 2020	September 2020–November 2020	December 2020–February 2021
Bagaciu	1	2	10	9
Bahnea	0	1	16	9
Band	4	1	56	36
Beica de Jos	0	2	20	6
Crăciunești	115	0	33	15
Faragau	1	6	8	6
Mica	0	4	13	14
Ogra	2	0	23	5
Petelea	0	1	21	17
Sanpaul	0	9	14	16
Vanatori	0	0	36	11
Viisoara	0	0	2	0
Zagar	0	0	8	3

**Table 2:** Statistics indicators of SAR-COV2 infestation in Mureș County.

	March 2020–May 2020		June 2020–August 2020		September 2020–November 2020		December 2020–February 2021	
	Villages with More than 25% *Țigani*	Villages with Less than 25% *Țigani*	Villages with More than 25% *Țigani*	Villages with Less than 25% *Țigani*	Villages with More than 25% *Țigani*	Villages with Less than 25% *Țigani*	Villages with More than 25% *Țigani*	Villages with Less than 25% *Țigani*
N (%)	123 (27.70%)	321 (72.29%)	26 (8.97%)	264 (91.03%)	260 (2.68%)	9416 (97.32%)	147 (2.87%)	4972 (97.13%)
Mean	9.46	3.61	2.00	2.97	20	105.80	11.31	55.87
SD	31.73	23.25	2.769	12.95	14.56	439.32	9.077	248.24
Maxim	115	219	9	112	56	4090	36	2313
95% CI	−9.714–28.64	−1.291–8.504	0.3268–3.673	0.2392–5.693	11.20–28.80	13.25–198.3	5.822–16.79	3.573–108.2

**Table 3:** Identifying statistical significance of SAR-COV2 infestation in Mureș County for villages with more *Țigani* vs. villages with less *Țigani*.

Period	Villages with More than 25% *Țigani* vs. Villages with Less than 25% *Țigani*
March 2020–May 2020	*p* = 0.8402
June 2020–August 2020	*p* = 0.2170
September 2020–November 2020	*p* = 0.1340
December 2020–February 2021	*p* = 0.1532
